# Practical Hints

**Published:** 1860-07

**Authors:** A. Hill


### ARTICLE IV.

### *Practical Hints.* By A. HILL, D. D. S.

#### *Expertness in Operating.*

"SLOW and sure," is a sound and useful maxim, but it is not always applicable, to dental operations.

It is a great mistake to suppose, that all operations in dentistry are valuable in proportion to the time consumed in performing them. Neither is it wise, on the other hand, to hurry matters too fast. It is sometimes true, that "haste, makes waste." It is often so indeed, in our profession. It is possible to err, on either hand. In avoiding *Scylla*, let us always look out for *Charybdis*.

Perhaps, as a general remark, we might say, with much propriety, that *hasty* dental operations, are the curse of the profession. But by this, we mean *careless* operations. They are not necessarily synonymous. But if by hasty operations, we mean superficial—and imperfect, showing neglect, and want of due care and attention, then, they are to be unhesitatingly condemned. This kind of practice is not what we refer to in our caption. We only mention it, to guard the thoughtful reader, against such a suspicion.

But there is an *expertness*—a *rapidity* in operating, which we especially commend, and concerning which, we propose to offer a few suggestions. And in doing this, we shall assume, and seek to illustrate, the following proposition, viz. There are circumstances in which an operation must be done *speedily*, or *rapidly*, if it is *well* done. To be *slow* in such a case, is not to be *sure* of success, but sure of failure.

We speak now, with direct reference to the operation of stopping teeth, although the same general remarks might be appropriately made, with reference to many other operations. Other things being equal, a surgeon who can operate most expeditiously, is the one to be preferred. And we have yet to learn, the necessity of detaining a patient under our hands for the space of three or six hours, in order to accomplish the filling of an *ordinary* cavity, where a little dexterity would complete the same in one hour.

We sometimes hear of dentists spending six or eight hours over *one* filling, and the time spent in such a case is referred to as evidence of superior care and pains-taking, and as affording a guarantee of superior workmanship. This is all well, and perhaps essential, in certain cases, but such cases come not within the scope of our present observations.

There are certain essential conditions to be met in every successful case of filling. And these conditions *must* be met, or failure will inevitably ensue. We will instance some of them.

1st. The cavity must be thoroughly cleaned.

2d. The walls must be adequate to retain the filling.

3d. The cavity must be entirely dry, when the filling is put in, and as far as possible, kept dry until finished.

4th. The filling must be densely packed, and remain in this condition, so as effectually to exclude *moisture* and *air*.

5th. The surface should be left sound and smooth.

Several other points might be named, but these are the great essentials. And not one of them can be neglected with impunity.

And now the great point is, whether in certain cases, we cannot best achieve the fulfilment of all these conditions, by an expeditious rather than by a slow and moderate method of manipulation. Or indeed, whether we can fulfil them at all except by the utmost dexterity. The circumstances under which we operate are exceedingly various. Some teeth are easy of access, while others are just the reverse. Some patients are fearless and calm, while others are timid and excitable. Some have large, some have small mouths. Some jaws are finely expanded and elliptical, while others are contracted and angular. The labial muscles can be easy pressed back in some cases, while in others the effort will make one's fingers ache to hold them even in moderate traction. Some mouths are comparatively dry, while others overflow, greatly to the annoyance of the dentist. Sometimes we have a clear sky, and a fine light, (which is very essential,) while at other times it is cloudy and dark, and we see with difficulty. Sometimes we are in good working condition, while at other times we are weary and exhausted.

It therefore becomes a question of serious importance to the dentist, how he may fulfil his duty, under the varied and trying circumstances of his practice.

However desirable it is, to bring to our operations the freshness and vigor, which is so requisite to success, as well as our own comfort, it is simply impossible in a large proportion of cases.

The confinement and tension of mind and body, is often so great, as, (in a little while, through inability to maintain it,) to disqualify us for delicate and precise manipulation.

Under such circumstances, with restless patients in waiting, where a prior engagement must be met, we often drag ourselves to our painful task, with a feeling of utter physical prostration. If, on the other hand, we come to our task with freshness, and even zest, our success in some measure depends upon the patient, notwithstanding all our skill.

There are certain conditions on their part to be fulfilled, else the results at which we aim, can never be fully realized.

We will enumerate a few of them, directly bearing upon our subject.

1st. The patient must be quiet—he must hold his mouth open, and hold it still.

2d. The patient must not interfere with the operator's hands.

3d. The patient must allow the dentist to prepare the cavity in the best possible manner, &c., &c.

The conditions named above are essential to success. Yet who does not know, that in a multitude of cases, they are most vexatiously interfered with? Sometimes indeed, they may all exist, and yet only exist for a short time, and if the operation is performed at all, it must be done with the greatest dispatch.

Under such circumstances, *rapid manipulation* is the only pledge of success.

No circumstances are more trying, and none furnish a severer test of the operator's skill. Linger a little, and the mouth overflows with saliva, washing the cavity, filling and all. Linger a little, and the patient becomes restive under your hands, until, together with mental anxiety, awkwardness of position, and tension of muscles, you lose the power of precise manipulation, and your operation fails. Amid such discouragements, you retire from the patient—wipe the sweat from your brow, and either abandon the case in despair, or allow the patient to leave your office, with the painful conviction upon your own mind, that you have but poorly fulfilled your task, and have failed to render the services demanded of you.

We know of but one way to overcome difficulties of this description; and that is, *make every stroke tell and make them quick.*

Nor can we get rid of these cases. We *must* meet them. And what is more, we must do justice to them, or sink degraded in our own estimation. It will not do to pass them by, as though we did not see them. Nor recommend extraction, while a more skillful dentist might save them. We must achieve what is possible, or sink to an inferior grade in our profession. For these are just the cases, which more than others try the operator's metal. An important class of operations, must of necessity be performed for the young. And such cases, more than others, challenge our care and attention.

And their well known restlessness and excitability, absolutely forbid, in many cases, the slow and graduated movements, which in others are clearly admissable.

This class of patients present many trying circumstances. They cannot always be made to understand the importance of the necessary confinement—nor the thoroughness of the operation. Neither can they sustain the tension required in the muscles, to keep the mouth open long enough, for a protracted operation. The salivary ducts, stimulated by the sensitive dentine, pour forth a flood of water, sufficient to keep in requisition all the pumps for such cases made and provided. And if an intelligent dentist can go through with these cases, and feel that he has done his whole duty, he is most certainly deserving of a high reputation among his fellows.

And more especially, if the cavities are difficult of access, by reason of their situation in the posterior portions of the molar teeth, and there is at the same time, an unnatural contraction of the muscles of the mouth, either congenital or accidental, the real difficulties must be greatly intensified.

To us, it is perfectly clear, that what is done in such a case,

"It were well, that it were done quickly."

We speak not with reference to the *stage* of the disease, but the *time* consumed in performing the operations. Indeed, it must be done quickly or not at all. No doubt, many cases of this sort are disposed of in a summary manner by extraction, in order to relieve the dentist of his trouble, but this method of practice can hardly satisfy an honorable ambition for distinction in the dental profession. Especially where a dentist wishes to achieve success, where success is the highest test of skill.

No fixtures, such as *lip-holders*, *tongue-holders*, *syringes*, *mouth-pumps*, *napkins*, &c., can ever compensate for lack of skill in dexterous, rapid and precise manipulation.

To fill *some* cavities, under the best combination of circumstances possible, is difficult enough in all conscience. But when, as above stated, these circumstances are against you, it requires no common energy to overcome them.

There are certain positions of body which a dentist is sometimes obliged to assume in order to accomplish his work, which it is absolutely impossible for him to sustain for any considerable length of time. And in order to complete his task, he must do it *quickly*, or fail. The muscles flag, and refuse their office, where there is long continued tension.

Many a case, deeply impressed upon the mind of the thoughtful reader, will occur to him, which will forcibly illustrate these views.

Not long since, the writer was summoned to a neighboring town, to attend to the teeth of a poor invalid young lady, who for more than ten years has been the subject of a most acute and painful illness. She was then in a state of comparative convalescence, yet a confirmed and almost helpless cripple. A violent hip disease had greatly distorted her limbs, while the cords of her neck were so contracted as to draw the head toward the right shoulder.

She had regained her health so as to be placed in a rocking chair, and yet without power to accommodate her position to the convenience of the operator.

Delicate and feeble, with scarcely ability enough to change her position at all, her head drawn down on one side, requiring a position on the part of the operator of the most tedious and awkward kind, surely such a case is sufficiently formidable.

The parents of the young lady being in affluent circumstances, were desirous to afford her every advantage which their ample means could command.

Her teeth were to be cleaned—several needed filling and two or three molars required extraction. Several cavities, were found in the posterior portions of the back grinders and one or two upon the labial surface near the gums. All presenting an array of difficulties from which any man might be supposed instinctively to shrink.

Here, both from the position of the operator, and the condition of the patient, it was needful that the work be done in the best manner and in the *shortest* possible time. And this was demanded by all the circumstances of the case.

And without any pretence on the part of the writer, that he was enabled to meet all the necessities of the case in the most perfect degree, it is suffictent to say, that this class of cases, during a practice of over twenty years, has wrought in his mind the firm conviction, that no man is competent to grapple with *such* difficulties who does not possess a good degree of skill and dexterity in dental manipulation.

